# Supplementary material for: Exploring immersion through a fMRI-compatible multi-finger handheld haptic display
Source: PLoS One. 2026 Mar 27;21(3):e0343297. doi: 10.1371/journal.pone.0343297 (PMC13029785; doi:10.1371/journal.pone.0343297)
Supplement: Attachment — Submitted filename: Response to Reviewers.pdf [file pone.0343297.s001.pdf]

Revised Paper (PONE-D-25-19819)

Exploring Immersion Through a fMRI-Compatible Multi-Finger Handheld Haptic Display

Joonsub Byun, Joonseon Hwang, Yong-An Chung, Hyeonseok Jeong, Jooyeon Kim, Keehoon Kim

First of all, we would like to express our deep gratitude to the editor and the reviewers for their valuable comments on the submitted manuscript. In response to the reviewers' comments, we have revised the manuscript and addressed all the issues that the reviewers questioned and pointed out. Particularly, we have focused on

- including the additional fundamental literature
- adding more explain on fMRI result
- and discussing the limitations of the proposed approach.

Our detailed responses to specific questions each reviewer raised are listed, along with the indications of what and where revisions have been made in the manuscript. All the modifications of the manuscript are highlighted with red and blue color so that the reviewers can easily locate them.

We appreciate your valuable time and comments.

To Academic Editor

Comment 1.

To resolve Reviewer #1's Comment #4, I would like you to cautiously or multidimensionally discuss the functionality of the identified brain regions. Please provide additional literature support that justifies your experimental paradigm and clearly outlines potential error ranges, emphasizing the consistency of BOLD signal changes.

Response 1.

Thank you for your comment. In response to the points raised, we would like to respectfully direct you to our detailed answer to **(R1.4)**, where we have thoroughly addressed these issues.

Comment 2.

Page 3: *In Table 1, please replace "O" and "X" with clear, universally understandable terms such as "yes/no" or "with/without," to avoid ambiguity for readers from diverse linguistic and cultural backgrounds.*

Response 2.

We sincerely thank the reviewer for this important suggestion. We agree that using "O" and "X" can be ambiguous for a global audience. Accordingly, we have replaced all instances of "O" with "Yes" and "X" with "No" throughout Table 1 to ensure clarity. The revised Table 1 can be found on page 3.

Comment 3.

Page 10: *Please remove the numbering "0.1" from the subsection titled "Experimental Evaluation: Haptic Display in VR with fMRI Results" under the Results section.*

Response 3.

Thank you for catching this formatting error. The numbering "0.1" was an unintentional artifact and has been removed from the subsection title. The corrected heading now appears on page 11.

Comment 4.

Figure 7 (b): *Adjust labels so they do not overlap or obscure the plotted curves.*

Response 4.

We appreciate the feedback regarding the clarity of Figure 7(b). In response, we have revised the figure to ensure the labels no longer overlap with any data curves. The updated Figure 7 is provided below.

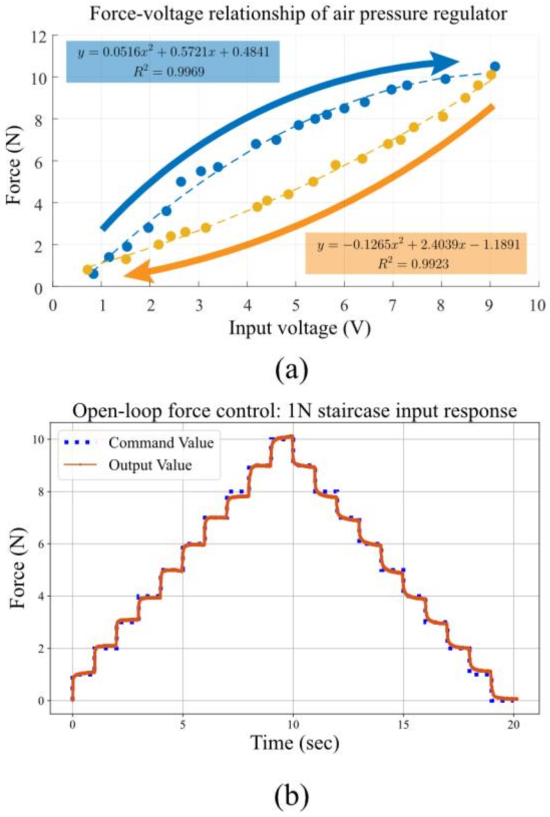

Comment 5.

Figure 9: Clearly explain the meaning of bins and the error bars (whiskers) in the figure legend or descriptive text accompanying the figure.

Response 5.

Thank you for highlighting the need for a more detailed explanation for Figure 9. We have revised the figure caption to explicitly define the components. The caption now states that "The box represents the interquartile range (IQR), showing the central 50\% of the data. The whiskers extend to the smallest and largest values within 1.5 times the IQR from the lower and upper quartiles, respectively." The revised caption is on page 10.

**Previous manuscript**
Box plot of tSNR for fMRI-compatibility test in different experimental conditions. (a) phantom tests with haptic display, and (b) human subjects tests with haptic display
**Revised manuscript**
Box plot of tSNR for fMRI-compatibility test in different experimental conditions. The box represents the interquartile range (IQR), showing the central 50\% of the data. The whiskers extend to the smallest and largest values within 1.5 times the IQR from the lower and upper quartiles, respectively. (a) phantom tests with haptic display, and (b) human subjects tests with haptic display

Response To Reviewer 1.

(R1.1)

1. The theoretical link between "immersion" and the experimental paradigm is unclear:

(R1.1.1)

The manuscript does not sufficiently discuss the dimensions that contribute to immersive experiences. Most cited studies focus on tactile feedback, but neglect other widely recognized components in VR research such as visual, auditory, motor, and bodily presence factors. The assumption that "tactile feedback = immersion" is theoretically unsubstantiated. Please provide a more comprehensive review of the literature and justify why tactile feedback alone can represent or significantly influence immersion. Why is touch prioritized as the dominant modality in this context?

(A1.1.1)

Thank you for your valuable observation. We did not intend to overlook the importance of visual, auditory, motor, and bodily presence factors in VR immersion. Rather, our study specifically focused on investigating the effect of haptic feedback on immersion. To address this insufficient explanation, we have added a comprehensive description of the various factors contributing to immersion in the Introduction section of the manuscript.

**Previous manuscript**
**Revised manuscript**
Several key factors contribute to VR immersion, including visual fidelity, spatial audio, interactivity, and presence. Among these, haptic feedback is particularly crucial because it completes the sensory experience by providing tactile sensations that correspond to virtual interactions. Haptic display is an interface aiming to enable bilateral signal communications between human and computer, and thus to greatly enhance the immersion and interaction of VR systems [3]. Since current consumer VR experiences are predominantly generated through visual and auditory feedback [4], incorporating haptic elements addresses a critical gap by engaging the sense of touch, creating more complete and compelling virtual experiences.<br>While researchers are indeed dedicated to enhancing the immersive experience through haptic technologies, the challenge of accurately evaluating this enhanced immersion persists. Since true immersion leads to a more engaging and realistic experience, a precise assessment of it is imperative.

(R1.1.2)

fMRI captures BOLD signal changes, but the link between these neural signals and subjective immersion is not well established. How can you ensure that the observed brain activations reflect

"immersion" rather than attention, task load, or movement-related factors?

(A1.1.2)

We appreciate the critical point regarding the challenge of isolating "immersion" from related cognitive states like attention, task load, or movement-related factors when interpreting BOLD signal changes. We agree that the direct link between neural signals and subjective immersion is complex and not fully established.

While our primary goal was to use fMRI to objectively quantify the neurological underpinnings of immersion, we acknowledge the potential for confounding variables. To mitigate these concerns and enhance the likelihood that our observed brain activations reflect aspects of immersion, we employed a meticulously designed experimental paradigm to control for extraneous variables.

In both experiments, participants performed the same core task, ensuring that general attention and task load were kept as consistent as possible across conditions. This allowed us to specifically isolate the contribution of haptic feedback to neural activity, beyond just general task engagement.

We recognize that in the first experiment, the presence or absence of haptic feedback might inherently lead to differences in physical load applied to the fingers. To address this, our second experiment was specifically designed to investigate brain activation when the physical load on the fingers remained constant, but the timing of the haptic feedback was varied

We acknowledge that fMRI results are not definitive, isolated indicators of subjective immersion. Instead, we interpret our findings as neural correlates of immersion-related processing and quantitative indicators of brain activity that contributes to the overall immersive experience. Immersion is a high-level cognitive and emotional state arising from the interplay of various brain functions. Our study aimed to reveal which neural mechanisms are modulated by haptic feedback in a way that contributes to this experience.

Our study aimed to quantitatively explore the impact of haptic feedback on immersion, based on M. Slater's definition of immersion as "the objective level of sensory fidelity." Following this approach, we focused our analysis of haptic feedback-related brain activation on two key aspects:

Multisensory Integration: We investigated how haptic feedback integrates with other sensory information (visual, auditory) to enhance overall sensory fidelity. We hypothesized that robust multisensory integration would lead to stronger neural activation in the sensory modalities involved in the experience. This serves as a direct mechanism for increasing the 'objective sensory fidelity'[1] of a virtual environment, thereby boosting immersion.

Higher-order Cognition: We sought to confirm whether haptic feedback, beyond simple sensory processing, interacts with higher-order cognitive processes crucial for the quality and depth of the immersive experience, such as attention, motor planning, and cognitive control. These cognitive aspects contribute to how effectively users process the sensory information provided by the system and interact with the environment, ultimately determining the level of immersion.

Consistent with these analytical foci, the observed brain activations across both experiments provided results for both multisensory integration and higher-order cognition.

In the first experiment, activations were observed in sensory-motor regions including the cerebellum, SMA, Caudate, Precentral Gyrus (PCG), Frontal Eye Field (FEF), Premotor Area (PMA), Primary Motor Cortex (M1), dorsolateral Prefrontal Cortex (dlPFC), and Primary Somatosensory Cortex (S1).

---

[1] M. Slater. note on presence terminology. Presence connect, vol. 3, no. 3, pp. 1–5, 2003.

These activations reflect direct sensory processing and motor responses to haptic feedback, along with the accompanying higher-order cognitive control.

The second experiment revealed activations in higher-order visual and integrative areas such as the anterior Prefrontal Cortex (aPFC), dlPFC, FEF, Parieto-occipital (PO) sulcus, Lingual Gyrus (LING), Cerebellum (CB), and Middle/Inferior Temporal Gyrus (MTG/ITG). This suggests that variations in haptic feedback timing influence visual information processing and cognitive integration, again involving higher-order cognitive control.

In summary, the activation areas observed across both experiments, including the consistent involvement of regions like dlPFC, FEF, and the cerebellum, alongside the specific sensory-motor regions in the first experiment and higher-order visual/integrative areas in the second, align with neural networks implicated in multisensory integration, attention, motor planning, and cognitive control. All of these are crucial components contributing to a comprehensive immersive experience. This alignment supports our research objective of providing quantitative neurological evidence for how haptic feedback enhances the objective aspect of immersion, as defined by M. Slater.

(R1.2)

2. Lack of direct evidence that tactile feedback enhances immersion:

(R1.2.1)

The study does not include behavioral data such as questionnaires or ratings from participants about their sense of immersion. This is a major weakness.

Please consider adding subjective measures or explain why they were not collected. Also discuss whether brain activity alone can be used as a reliable sign of immersion.

(A1.2.1)

Thank you for your valuable feedback regarding the lack of explicit subjective measures for immersion. We appreciate this opportunity to clarify our methodological choices and discuss the role of brain activity as an indicator of immersion.

Our primary objective was to deeply explore the neurological underpinnings of immersion experiences induced by haptic feedback. While quantifying the complex experience of immersion was a significant goal, we carefully considered the most appropriate methodology. We determined that self-report subjective measures could be prone to bias from participants' conscious cognition. Specifically, questionnaire items directly evaluating the impact of certain stimuli on immersion carried the risk of influencing participants to infer our research intentions and provide expected responses. We believed such potential biases could distort the pure brain responses captured by objective physiological indicators. Therefore, we opted to use fMRI data to minimize participant cognitive interference and focus on capturing the primary, often unconscious, effects of tactile feedback on brain activity. This approach allowed us to more clearly understand the mechanisms underlying the experience of immersion.

Regarding whether brain activity alone can be a reliable sign of immersion, we acknowledge that subjective experience is multifaceted and can't be fully encapsulated by neural data alone. However, fMRI provides objective and unbiased insights into the brain processes that underpin components of immersion, such as attentional modulation, sensory integration, and emotional processing. While not a direct measure of subjective feeling, observed modulations in brain regions associated with these functions serve as neural correlates contributing to immersion. Our focus was on identifying these underlying mechanisms.

We agree that integrating subjective measures would offer a more comprehensive understanding. As such, we plan to complement objective and subjective measures in future research to comprehensively investigate how these observed brain activity changes connect with actual subjective immersion experiences. This commitment is now explicitly stated in the discussion section of our manuscript.

**Previous manuscript**
Future work will incorporate position control and express texture using the piezo disk mentioned in the component compatibility test. The question of how immersive VR experiences will be enhanced by the fMRI-compatible multi-finger haptic display combined with texture feedback remains open, suggesting the possibility of extending beyond multi-finger to a multi-modal domain.

Revised manuscript
A primary limitation of the present study is the absence of subjective behavioral data, such as participant ratings on their sense of immersion. This was a deliberate methodological choice driven by our goal to investigate the neural underpinnings of immersion using objective and quantitative data. We were concerned that qualitative, self-report measures, such as questionnaires directly asking about the effects of stimuli, would carry a high risk of demand characteristics—prompting participants to infer our research hypothesis and provide biased responses. As such biases could confound the quantitative interpretation of the pure brain responses we aimed to capture, we prioritized observing the primary effects of haptic feedback on neural activity without this interference.<br><br>…<br><br>Future research should employ a multi-modal approach, combining objective fMRI data with subjective measures like validated presence questionnaires. This will be essential to comprehensively map the relationship between the neural correlates observed here and the subjective experience of immersion.<br>In addition to these methodological expansions, our future technical roadmap involves significant hardware advancements. We will incorporate position control and express texture using the piezo disk mentioned in the component compatibility test. The question of how immersive VR experiences will be enhanced by the fMRI-compatible multi-finger haptic display combined with texture feedback remains open, suggesting the possibility of extending beyond multi-finger to a multi-modal domain.

(R1.3)

3. Concerns about statistical methods:

(R1.3.1)

The fMRI analysis uses an uncorrected threshold ($p < 0.005$). In addition, the first brain image figure uses a cluster size threshold of 20 voxels, while the second uses 30 voxels. The reason for this difference is not explained.

Were these thresholds chosen using data-driven methods? Or were they selected without clear rules?

(A1.3.1)

We appreciate the reviewer's careful attention and for pointing out the inconsistency in the cluster size thresholds displayed in our fMRI figures. We sincerely apologize for this oversight.

To clarify, all fMRI analyses were consistently performed using a cluster size threshold of 25 voxels. This was explicitly stated in the Methods section (page 12 of the original manuscript).

The appearance of "20 voxels" in Figure 1 and "30 voxels" in Figure 2 was due to a simple error in the graphic labeling during figure generation, and not a reflection of a change in our analytical thresholds or a data-driven selection process for these specific figures. The 25-voxel threshold was chosen to balance sensitivity and specificity, given our sample size and the exploratory nature of the study.

We have now corrected Figures 12 and 13 to accurately reflect the consistent 25-voxel cluster size threshold used for all fMRI results.

(R1.3.2)

Using different and uncorrected thresholds increases the chance of false positives and creates concern about flexibility in analysis. Also, there is no correction for multiple comparisons (such as FWE or FDR), and no corrected p-values are reported. This does not meet current standards for fMRI analysis (e.g., Eklund et al., 2016, PNAS).

(A1.3.2)

We appreciate the reviewer's careful attention to detail regarding our fMRI analysis thresholds and the absence of multiple comparisons correction. We fully understand the critical importance of these methodological aspects for robust findings, as highlighted by Eklund et al. (2016) and current field standards.

Our decision to employ an uncorrected threshold was primarily necessitated by our small sample size. We acknowledge that this approach increases the chance of false positives and creates concern about flexibility in analysis. Therefore, we agree that our findings should be interpreted as exploratory. We have clarified this limitation within the manuscript to ensure appropriate interpretation of our results.

(R1.3.3)

The sample size is small (13 and 7 participants), so results should be interpreted with caution.

(A1.3.3)

Thank you for your valuable feedback. We have added a section to the Discussion addressing the limitations of our study due to the small sample size.

**Previous manuscript (for R1.3.2 ~ R1.3.3)**
**Revised manuscript (for R1.3.2 ~ R1.3.3)**
We acknowledge that the relatively small sample sizes (n=13 for group A and n=7 for group B) are a significant limitation of this study. While our findings offer initial insights, the limited number of participants constrains the generalizability of our results to a broader population. Therefore, any statistically significant findings should be interpreted with caution. Despite these limitations, this study serves as a crucial pilot investigation, successfully generating hypotheses for future research. Our haptic experiments provided valuable preliminary data, offering detailed insights into quantifying immersion.

(R.1.4)

4. Interpretation of fMRI results may be overstated:

(R1.4.1)

The activation of regions such as the dlPFC and FEF is more commonly associated with attention and task control, not immersion per se. The manuscript should offer a more careful discussion and avoid over-interpreting these activations.

(A.1.4.1)

We agree that the activation of regions like the dorsolateral prefrontal cortex (dlPFC) and the frontal eye fields (FEF) is commonly associated with attention and task control. These areas are indeed central to cognitive control and the allocation of attentional resources.

We interpret this activation not as 'immersion' per se, but as a reflection of the high-level cognitive control required to manage and facilitate the complex process of multisensory integration that underpins an immersive state. In such a state, frontal regions like the dlPFC and FEF exert top-down control to direct attentional resources, binding visual, auditory, and haptic information into a single, coherent experience. Specifically, as participants become more deeply immersed in a task, the demands for sustained attention, suppression of external distractions, and goal-directed behavioral control may intensify. This increased cognitive effort and regulation would naturally manifest as heightened activity in areas such as the dlPFC and FEF.

Therefore, we'll explicitly clarify that while dlPFC and FEF activity doesn't solely represent 'immersion' per se, it likely reflects the heightened attentional and cognitive control demands that accompany an immersive state. This will help us avoid over-interpretation and ensure a more precise discussion.

Previous manuscript
The cerebellum’s role in fine-tuning movement [29] and the FEF’s role in gaze control [30] were particularly noted, while the dlPFC showed activation related to attention [31] and task monitoring [32]
**Revised manuscript**
The cerebellum’s role in fine-tuning movement [29] and the FEF’s role in gaze control [30] were particularly noted, while the dlPFC showed activation related to attention [31] and task monitoring [32]. We interpret this activation as a reflection of the high-level cognitive control required to bind multiple sensory streams into a coherent immersion. Therefore, while not a direct measure of immersion, this activity represents the heightened attentional and cognitive demands essential for sustaining such a state.

(R1.4.2)

The synchronous vs. asynchronous task design may introduce confounding factors, such as violation of temporal expectation, which could activate regions like the SMG due to error awareness or attentional shift—not necessarily reduced immersion.

(A.1.4.2)

This is a very insightful point, and we appreciate the opportunity to clarify our interpretation. We agree that the asynchronous condition introduces a violation of temporal expectation. However, we contend that this is not a confound, but rather the core experimental manipulation designed to probe the mechanisms of immersion.

Our central theoretical premise is that immersion, as defined by M. Slater as 'objective sensory fidelity,' relies on the seamless integration of multisensory inputs. Temporal synchrony is a critical principle governing this integration. The asynchronous condition intentionally induces a temporal mismatch between sensory information—a 'sensory-motor conflict.' The 'violation of expectation' that the reviewer points out is, therefore, another expression of this conflict.

The supramarginal gyrus (SMG) is a critical node for detecting such visuomotor and sensorimotor mismatches[2] a process fundamental to both multisensory integration and the sense of agency. Accordingly, we interpret the differential activation in the SMG not as a simple 'error signal' independent of immersion, but as the neural correlate of the very failure of coherent multisensory integration that degrades objective sensory fidelity and consequently diminishes the immersive experience. To clarify our interpretation, we have now incorporated this concise explanation into the Discussion section of the manuscript.

Previous manuscript

[2] M. Tsakiris, M. R. Longo, P. Haggard P. Having a body versus moving your body: neural signatures of agency and body-ownership. Neuropsychologia. 2010 Jul;48(9):2740-9. doi: 10.1016/j.neuropsychologia.2010.05.021. Epub 2010 May 25. PMID: 20510255.

In contrast, the Asynchronous > Synchronous analysis revealed increased activation in the right SMG. Previous research suggests that increased the SMG activity is associated with awareness errors. Therefore, the results could be interpreted as possibly resulting from awareness errors between audiovisual feedback and proprioception.

Revised manuscript

In contrast, the Asynchronous > Synchronous analysis revealed increased activation in the right SMG. Previous research suggests that increased the SMG activity is associated with awareness errors. We propose that the asynchronous condition was designed to intentionally induce a 'sensory-motor conflict,' thereby disrupting the coherent multisensory integration that underpins the immersive experience. Therefore, the observed differential activation in the SMG is interpreted not as a simple error signal, but as the neural correlate of this failed integration, a process for which the SMG is known to be critical [43]. This failure of integration, which may include the awareness of errors between feedback modalities, ultimately degrades the objective sensory fidelity and diminishes the immersive experience.

(R1.5)

5. Additional suggestions:

(R1.5.1)

Please discuss whether the proposed experimental paradigm is generalizable to other types of VR tasks or user interactions.

(A1.5.1)

We thank you for this insightful point, which has given us the opportunity to further elaborate on our experimental paradigm. Accordingly, we have now integrated this discussion into the manuscript's Discussion section.

Previous manuscript

Revised manuscript

While the present study focused on a trumpet performance task, our experimental paradigm is broadly applicable to any rich multisensory scenario where haptic feedback from physical interaction occurs in concert with congruent audiovisual cues. This includes applications such as studying fine force interactions during keyboard typing, or providing tactile feedback for virtual object surface recognition, where the interplay between seeing, hearing, and feeling is critical.

(R1.5.2)

If possible, provide examples or preliminary data showing the applicability of this haptic device in different immersive scenarios to support its broader relevance.

(A1.5.1)

We agree that this would significantly strengthen the manuscript. As per your suggestion, we have expanded the Discussion section to include a detailed exploration of potential applications for our device.

**Previous manuscript**
**Revised manuscript**
Our haptic device, which provides force feedback to four fingers, adds a sense of realism to various scenarios. For example, users can directly feel with their fingertips the pressure of pressing a virtual button, the tension of drawing a bowstring, and the difference in stiffness between a soft sponge and a hard brick.

(R1.5.3)

Figures are clear, the manuscript is well-structured, and the overall language is fluent. However, some technical sentences are overly long and may benefit from simplification for better readability.

(A1.5.3)

We sincerely thank you for your valuable feedback, which helps to improve the quality of the manuscript. Reflecting your comments, we have revised and supplemented some of the rather long and complex technical sentences, as shown below, to make them easier for readers to understand.

**Previous manuscript**
The construction begins with the application of silicon gaskets on both sides of a 100 µm thick PET film, placed between acrylic plates of 2 mm, 3 mm, and 4 mm thicknesses to reduce friction and ensure sealing.
**Revised manuscript**
The module is constructed using a multi-layer assembly method. First, silicon gaskets are attached to both sides of a 100 µm-thick PET film. This film is then placed between acrylic plates with thicknesses of 2 mm, 3 mm, and 4 mm. This layered design serves two purposes: to reduce friction during movement and to ensure airtight sealing.

**Previous manuscript**

The proposed haptic device requires a compact sensor due to its handheld form. Among small-sized sensors, there are fewer fMRI-compatible force sensors or sensors that can measure pressure within the chamber. In addition, position information measured with optical fiber has severe nonlinearity, making it difficult to use for accurate position control.

Revised manuscript

The selection of an open-loop control system was due to several design constraints inherent to a handheld, fMRI-compatible device. Specifically, the device requires compact sensors, but options that are simultaneously small and fMRI-compatible are rare. Although alternatives like fiber optic sensors exist, we found their position data showed significant nonlinearity, rendering them inadequate for the precise position control required in a closed-loop system.

Previous manuscript

Individual analyses compared WH and WD sessions to rest (WH > rest and WD > rest) and between WH and WD. In the 1st-level specify, the parameters of the design matrix were set in the order of WD play, WD rest, WH play, and WH rest. The contrast value indicating WD $>$ Rest was set as [1 -1], whereas for WH > Rest, it was set as [0 0 -1 1]. The contrast value for WH $>$ WD was set to [-1 1 1 -1], and for WD > WH, it was set as [1 -1 -1 1].

Revised manuscript

For the first-level individual analyses, we constructed a design matrix to compare the 'With Haptics' (WH) and 'With Data Glove' (WD) sessions against rest periods and against each other. The parameters in the matrix were ordered as follows: WD play, WD rest, WH play, and WH rest. Based on this structure, we then defined the specific contrasts. The contrast for 'WD > Rest' was specified as [1 -1 0 0], and the contrast for 'WH > Rest' was [0 0 1 -1]. To directly compare the two conditions, the contrast for 'WH > WD' was set to [-1 1 1 -1], while its inverse, 'WD > WH', was set to [1 -1 -1 1].

Response To Reviewer 2.

We would like to sincerely thank you for your thoughtful and constructive comments. Please find our detailed responses below.

(R2.1)
Given the magnetic field of an MRI, and based on Figure 1 provided in the manuscript, is using standard VR headsets feasible in this setup? Or is it a mirror projection? Also, is Fig. 1 part of the experiment setup? If affirmative, I recommend adding it to the methodology section.

(A2.1)
Thank you for pointing this out. Standard VR headsets cannot be used inside an MRI scanner due to the strong magnetic field. Although MRI-compatible VR headsets exist, our experiment utilized a mirror projection system instead. To clarify this setup, we have added an explanation to the Methodology section of the manuscript. Additionally, Figure 1 does indeed represent part of our experimental setup, and we have now explicitly referenced it in the Methodology section as you recommended.

(R2.2)
Additionally, the manuscript does not specify the method used for auditory feedback delivery, which is essential for assessing immersion and may be affected by the ambient noise produced by the fMRI scanner.

(A2.2)
We appreciate your valuable observation. While we could not eliminate the scanner noise, we employed an MRI-compatible pneumatic headphone system to deliver trumpet sound feedback to participants. This information has now been clearly added to the *Methodology* section.

**Previous manuscript**
To investigate the effect of our proposed haptic display on the realism and immersion of VR experiences, we developed VR content that simulates piccolo trumpet performance, as illustrated in Fig 10. This content was created using Unity and designed to be compatible with our multi-finger haptic display.
**Revised manuscript**
To investigate the effect of our proposed haptic display on the realism and immersion of VR experiences, we developed VR content that simulates piccolo trumpet performance, as illustrated in Fig 10. This content was created using Unity and designed to be compatible with our multi-finger haptic display. The virtual reality trumpet playing system enabled subjects to view a monitor screen positioned above their head through a mirror projection system, as shown in Fig 1. Trumpet sounds were delivered via pneumatic headphones. Both the monitor display and pneumatic headphones were MRI-compatible.

(R2.3)

Was the task open-ended, allowing users to play freely, or did it follow a structured format, such as guiding users through specific sequences, like rhythm games like Guitar Hero?

(A2.3)

Thank you for this important question. The task followed a structured, rhythm-game-like format similar to "Guitar Hero." Buttons to be pressed were randomly selected and displayed on screen at regular intervals, with participants trained beforehand to respond to these visual prompts. We have updated the manuscript to include this clarification. We monitored participants during the experiment to ensure correct task performance. However, since the primary goal was to induce concentration and immersion rather than evaluate task performance, we did not report success rates or performance metrics in our analysis.

**Previous manuscript**
As shown in the experimental protocol in Fig 11, subjects repeated two sessions consisting of "Play" and "Rest" blocks in both experiments. Each condition of each experiment occurred in one session, and the order of the sessions was randomized for each subject. A 120-second interval between sessions ensured no carryover of activation from the first to the second session. A "Rest" block, lasting for 20 seconds, involved no active tasks. During the "Play" block, subjects engaged in trumpet playing through finger movements, receiving visual, auditory, and haptic feedback. Each session included ten cycles of Rest/Play blocks.
**Revised manuscript**
As shown in the experimental protocol in Fig 11, subjects repeated two sessions consisting of "Play" and "Rest" blocks in both experiments. Each condition of each experiment occurred in one session, and the order of the sessions was randomized for each subject. A 120-second interval between sessions ensured no carryover of activation from the first to the second session. A "Rest" block, lasting for 20 seconds, involved no active tasks. During the "Play" block, subjects engaged in trumpet playing through finger movements, receiving visual, auditory, and haptic feedback. The trumpet performance task was implemented using a structured format in which participants responded to visually cued button sequences. Random sets of buttons were highlighted in red and presented at regular 4-second intervals throughout the task. Each highlighted button configuration remained visible for 3 seconds, followed by a 1-second inter-stimulus interval before the subsequent set appeared. Participants were instructed to press the corresponding buttons as they became highlighted on the display. Each session included ten cycles of Rest/Play blocks.

(R2.4)

Another suggestion for improving the manuscript is to present the underlying neural mechanisms or brain regions the authors expect to observe during the experimental trials. For instance, while the cerebellum is discussed later in the results, it is not mentioned in the introduction. Including such expectations earlier would help contextualize the study's goals and hypotheses.

(A2.4)

Previous manuscript
Therefore, our research aims to provide a basis for quantifying immersion by measuring the change in activation in the brain's sensory-related areas through fMRI, depending on the presence or absence of haptic feedback.
**Revised manuscript**
Therefore, our research aims to provide a basis for quantifying immersion by measuring changes in activation within the brain's key sensory and cognitive networks. Specifically, we hypothesize that immersion levels will correlate with modulations in regions related to: (a) sensorimotor functions, such as the motor cortex, somatosensory cortices, and cerebellum; (b) audiovisual processing, like the temporal lobes; and (c) higher-order cognitive functions, including attention and sensory integration, governed by the frontal lobe.
