# Supplementary material for: Exploring immersion through a fMRI-compatible multi-finger handheld haptic display
Source: PLoS One. 2026 Mar 27;21(3):e0343297. doi: 10.1371/journal.pone.0343297 (PMC13029785; doi:10.1371/journal.pone.0343297)
Supplement: Attachment — Submitted filename: Response_to_Reviewers_auresp_2.pdf [file pone.0343297.s002.pdf]

Revised Paper (PONE-D-25-19819)

Exploring Immersion Through a fMRI-Compatible Multi-Finger Handheld Haptic Display

Joonsub Byun, Joonseon Hwang, Yong-An Chung, Hyeonseok Jeong, Jooyeon Kim, Keehoon Kim

First of all, we would like to express our deep gratitude to the editor and the reviewers for their valuable comments on the submitted manuscript. In response to the reviewers' comments, we have revised the manuscript and addressed all the issues that the reviewers questioned and pointed out. Particularly, we have focused on

- re-analysis using a permutation test with a threshold of $p < 0.001$ (uncorrected).
- reinforcement of the fMRI analysis methodology.

Our detailed responses to specific questions each reviewer raised are listed, along with the indications of what and where revisions have been made in the manuscript. All the modifications of the manuscript are highlighted with red and blue color so that the reviewers can easily locate them.

We appreciate your valuable time and comments.

To Academic Editor

(R1)

Please rerun some analysis to clarify doubts of the reviewer. The results must be backed up with support from literature in the Discussion section. Please avoid any overstated conclusions rather report facts and limitations if any.

(A1)

As requested, we have re-analyzed our data and revised the manuscript. We have taken care to focus on the factual findings and their limitations, avoiding any overstated conclusions. The details of these changes are provided in our response to Reviewer 3 and within the revised manuscript itself.

Response To Reviewer 1.

(R1)

thank you for your detailed revisions and clarifications. From the perspective of fMRI methodology and interpretation, the current version is clear and well addressed. I only suggest that the exploratory nature of the study be more explicitly emphasized in the manuscript, as this is a crucial point for appropriately framing the findings.

(A1)

We are very grateful for the reviewer’s positive assessment and for their constructive guidance, which has been instrumental in improving our manuscript.

As indicated by our manuscript's title ("Exploring Immersion..."), our research was designed as an exploratory study from the outset. As suggested, we have now added explicit statements highlighting the study's exploratory nature in the sections of the Introduction where we outline the study's aims, as well as in the Discussion section to properly frame the interpretation of our findings.

Thank you once again for your valuable contributions to our manuscript.

**Previous manuscript (1) - Introduction**
Building on this foundation, our research objectives are as follows: (1) Developing a multi-finger haptic display that provides VR with immersive and fMRI-compatibility with fMRI. (2) Establishing the feasibility of quantitative measurement regarding user immersion based on VR content suitable for multi-finger haptic displays.
**Revised manuscript (1) - Introduction**
Building on this foundation, our research objectives are as follows: (1) Developing a multi-finger haptic display that provides VR with immersive and fMRI-compatibility with fMRI. (2) Exploring the feasibility of quantitative measurement regarding user immersion based on VR content suitable for multi-finger haptic displays.

**Previous manuscript (2) - Discussion**
These findings emphasize that haptic feedback increases sensory fidelity, thus improving immersion in VR tasks like trumpet performance.
**Revised manuscript (2) - Discussion**
These findings provide preliminary evidence that haptic feedback increases sensory fidelity, thus improving immersion in VR tasks like trumpet performance.

Response To Reviewer 3.

(R3)

The current manuscript uses questionable fMRI analysis methods that does not meet current fMRI analysis standards. This was raised by a previous reviewer, and I don't believe the authors have adequately addressed the concerns by putting this just as a limitation. I understand the limitations of sample size; however, this is not an adequate reason not to conduct fMRI analysis to modern analysis standards.

(A3)

We thank the reviewer for their valuable feedback and for raising this important point regarding our statistical analysis methods. We sincerely apologize that our previous revisions were insufficient.

In accordance with the reviewer's suggestion, we have now conducted a complete re-analysis of our fMRI data. Please find our detailed response below.

(R3.1)

Please redo the analysis with an uncorrected threshold of p < 0.001 (which is a standard uncorrected threshold measure). Then do an adequate correction for multiple comparisons (ideally permutation testing) for all the contrasts. If the authors achieve no significant results, for the contrast WH > WD in experiment one, I could maybe see an argument to present the uncorrected findings so long as there isn't speculation of neural mechanisms, but rather a discussion on the importance of haptic feedback.

(A3.1)

We thank the reviewer for this constructive suggestion.

As requested, we have re-conducted our second-level analyses using a more rigorous statistical approach. We first applied a standard cluster-forming threshold of $p < 0.001$ (uncorrected). Subsequently, we performed a robust correction for multiple comparisons for all contrasts using non-parametric permutation testing, as implemented in the SnPM toolbox. In this re-analysis, we also adjusted the cluster extent threshold to 20 voxels.

This new analysis yielded significant results. For the WH > WD contrast, we observed significant activation in the bilateral postcentral gyrus. For the Sync > Async contrast, significant clusters were found in several regions, including the cerebellum and lingual gyrus.

Activations were also observed in other regions, including the middle temporal gyrus, caudate, and precentral gyrus. However, these clusters are not reported as they did not meet the minimum size threshold of 20 contiguous voxels.

The full details of these new results have been added to the revised manuscript. We kindly ask the reviewer to refer to the revised manuscript for these changes.

As part of this revision, we have also reorganized the entire fMRI analysis methods section for improved clarity. The following change is an excerpt detailing the revisions made to the second-level analysis portion of our fMRI analysis methods.

Previous manuscript

Experiment 1: With or Without haptic feedback

…

fMRI Data Analysis

…

Group-level statistical analysis was conducted using one-sample t-tests on WH > WD and WH > WD contrasts. Direct comparisons between haptic display and data glove sessions were made by calculating the difference between WH > rest and WD > rest contrasts, applying two-sample one-sample t-tests for WH > WD and WD > WH contrasts. Significance thresholds were set to an uncorrected p-value of 0.005, ignoring clusters smaller than 25 voxels.

Experiment 2: Synchronous versus Asynchronous

…

fMRI Data Analysis

…

For aggregating results across all subjects, a statistical analysis was conducted using a one-sample t-test in the 2nd-level analysis. The uncorrected p-value was established at 0.005, and clusters containing fewer than 25 voxels were excluded from the analysis.

Revised manuscript

fMRI Data Analysis

For both experiments, the resulting contrast images from each subject were carried forward to a second-level, random-effects group analysis. A one-sample t-test model was used for each contrast, with statistical significance assessed using non-parametric permutation testing (5000 permutations) as implemented in the SnPM13 toolbox [29]. For cluster-wise statistical inference across the whole brain, a cluster-forming threshold of p $>$ 0.001 (uncorrected) was applied at the voxel level, and only clusters containing 20 or more contiguous voxels are reported.

(R3.2)

The current manuscript overstates fMRI findings and makes claims that are not supported by the analysis methods. Again, this was raised by a previous reviewer, and I do not feel the authors in the updated manuscript have adequately addressed this. Given the small sample size and the questionable fMRI statistical methods there is a high probability that findings are not robust and as such any talk of psychological/neural mechanisms to explain these findings is not currently appropriate. This may change once a more rigours analysis has been done. If it doesn't then a discussion on why results were not found is important instead of on possible mechanisms seems more appropriate.

(A3.2)

We thank the reviewer for this critical feedback. We agree that our initial interpretations were not adequately supported by the previous analysis.

As detailed in our response above (A3.1), we have now conducted a more rigorous analysis using a standard cluster-forming threshold ($p<0.001$) and non-parametric permutation testing for multiple comparisons correction.

In light of these new, more robust results, we have carefully revised the manuscript. We have reframed our discussion to be appropriately cautious and ensured our claims are well-supported by the data, avoiding any overstatement of the findings. The revised interpretations can be found in the updated manuscript.

**Previous manuscript**
The cerebellum, the FEF, and the dlPFC were activated in both WH > WD and Synchronous > Asynchronous.<br><br>The cerebellum's role in fine-tuning movement [29] and the FEF's role in gaze control [30] were particularly noted, while the dlPFC showed activation related to attention [31] and task monitoring [32]. We interpret this activation as a reflection of the high-level cognitive control required to bind multiple sensory streams into a coherent immersion. Therefore, while not a direct measure of immersion, this activity represents the heightened attentional and cognitive demands essential for sustaining such a state.<br><br>Additionally, WH > WD contrasts revealed stronger activation in motor-related areas like the contralateral M1 and S1, which are involved in spatiotemporal processing of multisensory information [34] and object manipulation [35]. Increased activation in the PMA and SMA highlighted their role in temporal coordination during motor control [36]. Cognitive regions like the PCG and Caudate were also more active when haptic feedback was present, reflecting enhanced perception and decision-making. The PCG responds to cognitive task states such as perceptual decisions and motor responses [37]. And the Caudate plays the role of reward-based decision making [38].<br><br>Synchronous > Asynchronous analysis also showed increased activation in brain regions related to higher-order cognition , including the MTG, ITG, PO, and LING, which are involved in the conceptual processing of tools [39], music processing [40], cognition [41], and task management [42]. This supports the idea that synchronized haptic feedback enhances immersion by engaging higher cognitive processes. In contrast, the Asynchronous > Synchronous analysis revealed

increased activation in the right SMG. Previous research suggests that increased the SMG activity is associated with awareness errors [43]. We propose that the asynchronous condition was designed to intentionally induce a 'sensory-motor conflict,' thereby disrupting the coherent multisensory integration that underpins the immersive experience. Therefore, the observed differential activation in the SMG is interpreted not as a simple error signal, but as the neural correlate of this failed integration, a process for which the SMG is known to be critical [44]. This failure of integration, which may include the awareness of errors between feedback modalities, ultimately degrades the objective sensory fidelity and diminishes the immersive experience.

While the present study focused on a trumpet performance task, our experimental paradigm is broadly applicable to any rich multisensory scenario where haptic feedback from physical interaction occurs in concert with congruent audiovisual cues. This includes applications such as studying fine force interactions during keyboard typing, or providing tactile feedback for virtual object surface recognition, where the interplay between seeing, hearing, and feeling is critical.

These findings emphasize that haptic feedback increases sensory fidelity, thus improving immersion in VR tasks like trumpet performance. Our multi-finger haptic device offers a valuable tool for quantifying immersion in VR and is adaptable for a range of tasks with multiple degrees of freedom.

Revised manuscript

The comparison between the presence and absence of haptic feedback (WH > WD) revealed stronger activation in the contralateral postcentral gyrus. This is consistent with its established role in integrating finger-specific motor and tactile information [30] and in object manipulation [31].

Furthermore, the Synchronous > Asynchronous analysis highlighted a network of regions involved in binding multisensory information into a coherent whole. Activation in the lingual gyrus (LING) aligns with its function in processing complex visual information [32]. The engagement of the fusiform gyrus is particularly noteworthy, as this region is associated with processing semantic congruency between haptic objects and their related sounds [33]. Moreover, the parahippocampal gyrus (PHG) activation is consistent with its specific role in encoding kinematic parameters, such as speed and direction, during visuomotor tasks. Together, this pattern suggests that synchronous feedback facilitates the integration of visual, haptic, and contextual spatial information into a meaningful percept.

The cerebellum's involvement can be attributed to its role in performance monitoring and temporal prediction [34,35], both of which are essential for synchronizing actions with sensory feedback in timed-response tasks. Crucially, the insula has been implicated in higher-order cognitive control and attention [36]. Several reports have noted increased insula activation as subjects became more aware of being in control of an action [37,38]. Also, its known involvement in stimulus-driven attention and self-awareness processes has been directly linked to the sense of presence [39].

These findings provide preliminary evidence that haptic feedback increases sensory fidelity, thus improving immersion in VR tasks like trumpet performance.

(R3.3)

More detail in the main manuscript is needed regarding fMRI modelling and results. At the pre-processing stage, please state exactly which steps were conducted and in what order.

At the first level models please put how the BOLD signal was modelled (i.e. which HRF, any derivatives etc) and if any filtering of the time series was conducted. Please provide details of confound regressors used in the model, such as head motion, and state how many regressors were used at this stage. Please also state how the temporal autocorrelation was modelled.

(A3.3)

We thank the reviewer for this valuable suggestion. We have clarified the details of our first-level analysis below.

For the first-level models, the blood-oxygen-level-dependent signal was modeled by convolving the event onsets with the canonical hemodynamic response function provided by SPM. To remove low-frequency noise and scanner drift, the time series data were high-pass filtered using a cutoff period of 128 seconds.

To account for nuisance variance, the model included several confound regressors. These were the six head motion parameters derived from the realignment stage, consisting of three translations and three rotations. A session-specific constant term was also included for each of the two sessions per subject. This resulted in a total of 14 confound regressors for each subject, comprising six motion parameters and one constant per session.

Finally, temporal autocorrelation was modeled using a first-order autoregressive model.

This detailed description has now been added to the Methods section of our revised manuscript.

**Previous manuscript**
The preprocessing pipeline included realignment to correct for head motion, spatial normalization to an East Asian brains ICBM space template, and smoothing with an 8-mm FWHM Gaussian kernel.
**Revised manuscript**
The preprocessing pipeline consisted of the following steps, performed in order: (1) realignment to correct for head motion, (2) spatial normalization to an East Asian brains ICBM space template, and (3) spatial smoothing with an 8-mm Full Width at Half Maximum (FWHM) Gaussian kernel.<br><br>First-level statistical analyses were performed for each subject. The BOLD signal was modeled using the canonical hemodynamic response function in SPM12. A high-pass filter with a 128-second cutoff period was applied to the time series, and temporal autocorrelation was modeled using a first-order autoregressive model. Six head motion parameters generated by the realignment in SPM12 and a session-specific constant were included as confound regressors for each of the two imaging runs, resulting in a total of 14 confound regressors per subject.

(R3.4)

For the second level models, it needs to be clearly stated whether this was a whole brain, or a ROI based analysis. Please present the t statistic, the contrast estimates, the corresponding p-value, the x, y, z co-ordinates along with the size for each significant finding. Please also state which atlas was used to determine location of regions. Ideally this should be done in a table.

(A3.4)

We thank the reviewer for their detailed feedback and for the clear guidance on how to improve the reporting of our second-level model results.

To address your comments, we would first like to clarify that our second-level models involved a whole-brain analysis. In accordance with your suggestions, we have compiled all significant findings into a new table which has been added to the manuscript.

This table now presents the key statistical parameters for each significant cluster, including the t-statistic, the corresponding p-value, cluster size (k), and the peak MNI coordinates (x, y, z).

Furthermore, we have added a statement clarifying that the anatomical locations of these regions were determined using the Automated Anatomical Labeling (AAL) atlas

**Previous manuscript**
**Revised manuscript**
**In Table 6**<br><br>Brain regions showing significant activation for the WH > WD (k > 20). Anatomical locations were identified using the Automated Anatomical Labeling (AAL) atlas.<br><br>**In Table 7**<br><br>Brain regions showing significant activation for the Sync > Async contrast (k > 20). Anatomical locations were identified using the Automated Anatomical Labeling (AAL) atlas.

(R3.5)

Limitations about behavioural data.
The justification for not using behavioural measures does not seem to be a valid objection. It is also contradictory as the authors argue about influencing results but then recommend using behavioural work in future research. Please remove the justification part of not using behavioural data. Then just put that a primary limitation of the present study does not use behavioural data, and that future work should use behavioural data.

Limitations should have its own subheading.

(A3.5)

We thank the reviewer for this insightful comment. We agree that our original justification for not including behavioral measures was contradictory.

In response, we have revised the manuscript to address this. We have created a dedicated "Limitations" section and removed the previous justification. The revised section now clearly states that the absence of behavioral data is a primary limitation and emphasizes that future work should incorporate such measures.

**Previous manuscript**
A primary limitation of the present study is the absence of subjective behavioral data, such as participant ratings on their sense of immersion. This was a deliberate methodological choice driven by our goal to investigate the neural underpinnings of immersion using objective and quantitative data. We were concerned that qualitative, self-report measures, such as questionnaires directly asking about the effects of stimuli, would carry a high risk of demand characteristics, prompting participants to infer our research hypothesis and provide biased responses. As such biases could confound the quantitative interpretation of the pure brain responses we aimed to capture, we prioritized observing the primary effects of haptic feedback on neural activity without this interference.<br><br>We acknowledge that the relatively small sample sizes (n=13 for group A and n=7 for group B) are a significant limitation of this study. While our findings offer initial insights, the limited number of participants constrains the generalizability of our results to a broader population. Therefore, any statistically significant findings should be interpreted with caution. Despite these limitations, this study serves as a crucial pilot investigation, successfully generating hypotheses for future research. Our haptic experiments provided valuable preliminary data, offering detailed insights into quantifying immersion.
**Revised manuscript**
**Limitations**<br><br>We acknowledge that the relatively small sample sizes (n=13 for group A and n=7 for group B) and using uncorrected p for analysis are a significant limitation of this study. Furthermore, a primary limitation of the present study is the absence of subjective behavioral data, such as participant ratings on their sense of immersion. While our fMRI results suggest potential neural correlates, future research is required to strengthen these findings by demonstrating a direct correlation between these brain activations and participants' self-reported experiences. Such studies will be essential to elucidate the precise mechanisms by which these neural patterns contribute to an enhanced sense of immersion and to comprehensively map the relationship between objective neural markers and the subjective feeling of immersion.

Despite these limitations, this study serves as a crucial pilot investigation, successfully generating hypotheses for future research. Our haptic experiments provided valuable preliminary data, offering detailed insights into quantifying immersion.

(R3.6)

In Table 4. fMRI-compatibility test result of the proposed haptic display
Please present p value for Haptic display on, 0 cm. Also, please indicate which p value the test applies (is it the post hoc or the Kruskal-Wallis)

(A3.6)

We apologize for this omission and thank you for pointing it out. The p-value for the "Haptic display on, 0 cm" condition was highly significant and is now reported as p<0.001. This value was derived from the post-hoc test.

To eliminate ambiguity, we have added notes to Table 4 that clearly specify whether a given p-value corresponds to the overall Kruskal-Wallis test or a specific post-hoc comparison. Thank you again for your constructive feedback.

**Previous manuscript**
In Table 4 table notes<br><br>Signal, noise and tSNR data were measured and averaged according to each ROI.<br>The p-value corresponds to either the Kruskal-Wallis test or the post hoc test.
**Revised manuscript**
In Table 4 table notes<br><br>Signal, noise and tSNR data were measured and averaged according to each ROI.<br>The p-value for 0cm condition corresponds to the post-hoc test.<br>The p-value for 67cm condition corresponds to the Kruskal-Wallis test.
