# Supplementary material for: Exploring immersion through a fMRI-compatible multi-finger handheld haptic display
Source: PLoS One. 2026 Mar 27;21(3):e0343297. doi: 10.1371/journal.pone.0343297 (PMC13029785; doi:10.1371/journal.pone.0343297)
Supplement: Attachment — Submitted filename: Response_to_Reviewers_auresp_3.pdf [file pone.0343297.s003.pdf]

Revised Paper (PONE-D-25-19819)

Exploring Immersion Through a fMRI-Compatible Multi-Finger Handheld Haptic Display

Joonsub Byun, Joonseon Hwang, Yong-An Chung, Hyeonseok Jeong, Jooyeon Kim, Keehoon Kim

First of all, we would like to express our deep gratitude to the editor and the reviewers for their valuable comments on the submitted manuscript. In response to the reviewers' comments, we have revised the manuscript and addressed all the issues that the reviewers questioned and pointed out. Particularly, we have focused on

- Description of cluster extent threshold

Our detailed responses to specific questions each reviewer raised are listed, along with the indications of what and where revisions have been made in the manuscript. All the modifications of the manuscript are highlighted with red and blue color so that the reviewers can easily locate them.

We appreciate your valuable time and comments.

Response To Reviewer 3.

(R3)

Can the authors clarify:

1) If they used cluster-based correction with a cluster forming threshold ($p<0.001$ uncorrected) and a cluster extent threshold (p corrected <0.05).

2) The cluster extent threshold is not defined by an arbitrary k, but on a corrected p value (p FWE/FDR <0.05) derived from the permutation tests.

> If the authors have based the cluster extent threshold on an arbitrary level ($k > 20$) rather than on a corrected p value from the permutation tests, then:
>
> 1) The authors must define the cluster extent threshold as a corrected (FWE/FDR) $p <0.05$ derived from the permutation tests.
>
> 2) Only clusters that survive this level of multiple comparisons correction should be reported.
>
> 3) I would encourage the authors to read the cluster failure paper (Eklud et al., 2016) which persuasively shows why using arbitrary cluster extents does not control the type I error rate.

(A3)

We sincerely thank the reviewer for their insightful comments and for identifying this critical issue in our statistical analysis. The reviewer is absolutely correct that our previous approach, which employed an arbitrary cluster extent threshold ($k > 20$), failed to meet current neuroimaging standards and did not adequately control for Type I error.

Following the application of the appropriate cluster-level FDR correction ($p < .05$), we found that no clusters survived this threshold in either experiment. We now explicitly acknowledge this null finding at the outset of the Results section and have completely removed the arbitrary $k > 20$ threshold and "significant" words from both our analysis and the manuscript.

To provide transparency and context for future research, we report results identified at the cluster-forming threshold ($p < .001$, uncorrected) for purely exploratory and descriptive purposes. Rather than applying an arbitrary threshold, we now report the four largest clusters for each contrast.

All corresponding figures and tables have been revised with explicit labels indicating that these findings did not survive multiple comparison correction (e.g., "all $p_{FDR} > .05$"), ensuring readers fully understand the exploratory nature of these results.

We believe these substantial revisions directly address the reviewer's concerns regarding statistical rigor and transparency. We are grateful for the reviewer's constructive feedback, which has significantly strengthened our manuscript.

**Previous manuscript (1) - Result**
**Revised manuscript (1) - Result**
No clusters survived the cluster-level FDR correction ($p < 0.05$) in either experiment. However, for exploratory purposes and to guide future works, we report the results identified using a cluster-forming threshold of $p < 0.001$ (uncorrected). For each contrast, the 4 largest clusters are reported for descriptive purposes (all p_{FDR} > 0.05).

**Previous manuscript (2) - Table 6**
Brain regions showing significant activation for the WH > WD (k > 20). Anatomical locations were identified using the Automated Anatomical Labeling (AAL) atlas.
**Revised manuscript (2) - Table 6**
Brain regions showing activation for the WH > WD contrast. Clusters were identified using a cluster-forming threshold of $p < 0.001$ (uncorrected). The 4 largest clusters are shown for exploratory purposes (all p_{FDR} > 0.05). Anatomical locations were identified using the Automated Anatomical Labeling (AAL) atlas.

**Previous manuscript (3) - Figure 13**
Neurological view corresponding to group fMRI results showing dominant activation regions in the WH > WD contrast (PG - Postcentral gyrus)
**Revised manuscript (3) - Figure 13**
Neurological view corresponding to group fMRI results showing dominant activation regions in the WH > WD contrast (PG - Postcentral gyrus, p_{FDR} > 0.05)

Previous manuscript (4) - Table 7

Brain regions showing significant activation for the Sync > Async contrast (k > 20). Anatomical locations were identified using the Automated Anatomical Labeling (AAL) atlas.

Revised manuscript (4) - Table 7

Brain regions showing activation for the Sync > Async contrast Clusters were identified using a cluster-forming threshold of p < 0.001 (uncorrected). The 4 largest clusters are shown for exploratory purposes (all p_{FDR} > 0.05). Anatomical locations were identified using the Automated Anatomical Labeling (AAL) atlas.

Previous manuscript (5) - Figure 14

Neurological view corresponding to group fMRI results showing dominant activation regions in the Synchronous $>$ Asynchronous contrast (LING - lingual gyrus, CB - Cerebellum, PHG - Parahippocampal Gyrus).

Revised manuscript (5) - Figure 14

Neurological view corresponding to group fMRI results showing dominant activation regions in the Synchronous $>$ Asynchronous contrast (LING - lingual gyrus, CB - Cerebellum, PHG - Parahippocampal Gyrus, all $p_{FDR} > 0.05)

Previous manuscript (6) - Result & Discussion

As illustrated in Fig 13, significant activation was observed in the left cerebellum and lingual gyrus (LING). Additionally, a large cluster was identified with its peak in the left white matter (MNI: -40, -12, 26), which extended inferiorly to encompass a portion of the left insula. In the right hemisphere, significant activations were found in the fusiform gyrus and the parahippocampal gyrus (PHG).

The fMRI experiments with VR trumpet performance highlight the effectiveness of our multi-finger haptic device in quantifying immersion. Significant increases in brain activity were observed when haptic feedback was present and synchronized with audiovisual cues.

Revised manuscript (6) - Result & Discussion

As illustrated in Fig 13, activation was observed in the left cerebellum and lingual gyrus (LING). Additionally, a large cluster was identified with its peak in the left white matter (MNI: -40, -12, 26), which extended inferiorly to encompass a portion of the left insula. In the right hemisphere, activations were found in the fusiform gyrus and the parahippocampal gyrus (PHG).

The fMRI experiments with VR trumpet performance highlight the effectiveness of our multi-finger haptic device in quantifying immersion. Increases in brain activity were observed when haptic feedback was present and synchronized with audiovisual cues.
