# Supplementary material for: Exploring immersion through a fMRI-compatible multi-finger handheld haptic display
Source: PLoS One. 2026 Mar 27;21(3):e0343297. doi: 10.1371/journal.pone.0343297 (PMC13029785; doi:10.1371/journal.pone.0343297)
Supplement: Attachment — Submitted filename: Response_to_Reviewers_auresp_4.pdf [file pone.0343297.s004.pdf]

Revised Paper (PONE-D-25-19819)

Exploring Immersion Through a fMRI-Compatible Multi-Finger Handheld Haptic Display

Joonsub Byun, Joonseon Hwang, Yong-An Chung, Hyeonseok Jeong, Jooyeon Kim, Keehoon Kim

First of all, we would like to express our deep gratitude to the editor and the reviewers for their valuable comments on the submitted manuscript. In response to the reviewers' comments, we have revised the manuscript and addressed all the issues that the reviewers questioned and pointed out. Particularly, we have focused on

- Description of cluster extent threshold

Our detailed responses to specific questions each reviewer raised are listed, along with the indications of what and where revisions have been made in the manuscript. All the modifications of the manuscript are highlighted with red and blue color so that the reviewers can easily locate them.

We appreciate your valuable time and comments.

Response To Reviewer 4.

(R4 -1)

The introduction reiterates "importance of immersion" multiple times. Can be reduced by ~25% without loss of content.

(A4 -1)

As suggested, I have condensed the Introduction section by removing repetitive descriptions regarding the 'importance of immersion'.

**Previous manuscript (1)**
For example, virtual reality can be used to train surgeons or firefighters using haptic devices and has cost and risk reduction advantages compared to reality [1, 2]. Immersion plays a pivotal role in VR applications, as it grants users a sense of `being' within the task environment, fostering a feeling of truly `being there.' This immersion is critical for delivering a realistic experience. Despite technological advances, maintaining immersion in the virtual environment, especially during physical interaction, remains a challenge due to users' sensitivity to the distinctions between the real and virtual worlds.
**Revised manuscript (1)**
For example, virtual reality can be used to train surgeons or firefighters using haptic devices and has cost and risk reduction advantages compared to reality [1, 2]. Immersion acts as the cornerstone of these applications, granting users a sense of `being' within the task environment. Despite technological advances, maintaining immersion in the virtual environment, especially during physical interaction, remains a challenge due to users' sensitivity to the distinctions between the real and virtual worlds.

**Previous manuscript (2)**
While researchers are indeed dedicated to enhancing the immersive experience through haptic technologies, the challenge of accurately evaluating this enhanced immersion persists. Since true immersion leads to a more engaging and realistic experience, a precise assessment of it is imperative. Previous studies have used qualitative methods, such as surveys, to evaluate VR immersion in relation to audio-visual [5] and haptic feedback [6]. However, the qualitative method entails problems, such as not being able to measure the immersion experienced by users in real time, and being unable to accurately express what state the users themselves are in. Therefore, quantitative research on immersion is necessary, but it is currently underdeveloped in this field.

Revised manuscript (2)
While researchers are dedicated to enhancing immersion through haptic technologies, accurately evaluating it remains a challenge. Previous studies have used qualitative methods, such as surveys, to evaluate VR immersion [5, 6]. However, qualitative methods are limited by their inability to measure user experience in real time or accurately express the user's state. Therefore, quantitative research on immersion is necessary but currently underdeveloped.

Previous manuscript (3)
However, most studies have primarily focused on the visual and auditory aspects of immersion [7], neglecting the critical role of haptic feedback. Haptic feedback, as important as visual and auditory elements, is necessary for creating a fully immersive VR experience [8]. It requires sensory feedback based on haptic interaction to enhance immersion, boosting the sense of presence by simulating physical interactions through realistic sensations [9].
**Revised manuscript (3)**
However, most studies have primarily focused on the visual and auditory aspects of immersion [7], neglecting the critical role of haptic feedback. Haptic feedback is a critical component of system immersion, as it heightens sensory realism—a key factor in eliciting the user's subjective sense of presence [8, 9].

(R4 -2)

"Immersion," "presence," and "realism" are sometimes used interchangeably. These are not equivalent constructs in VR literature.

(A4 -2)

Except for the introduction, the word "presence" in this paper is used in the context of the "presence" of haptic feedback (device). Therefore, the part in the introduction that seemed to be confused with "immersion" has been revised to clarify the relationship between immersion and presence.

**Previous manuscript (4)**
However, most studies have primarily focused on the visual and auditory aspects of immersion [7], neglecting the critical role of haptic feedback. Haptic feedback, as important as visual and auditory elements, is necessary for creating a fully immersive VR experience [8]. It requires sensory feedback based on haptic interaction to enhance immersion, boosting the sense of presence by simulating physical interactions through realistic sensations [9].
**Revised manuscript (4)**
However, most studies have primarily focused on the visual and auditory aspects of immersion [7], neglecting the critical role of haptic feedback. Haptic feedback is a critical component of system immersion, as it heightens sensory realism—a key factor in eliciting the user's subjective sense of presence [8, 9].

As you pointed out, the word "Realism" seems to have been used interchangeably. To address this, I've revised the following sentence.

**Previous manuscript (5)**
To investigate the effect of our proposed haptic display on the realism and immersion of VR experiences, we developed VR content that simulates piccolo trumpet performance, as illustrated in Fig 10.
**Revised manuscript (5)**
To investigate the effect of our proposed haptic display on the immersion of VR experiences, we developed VR content that simulates piccolo trumpet performance, as illustrated in Fig 10.

Realism, which appears elsewhere, is not used interchangeably with immersion, but rather in the sense that it provides a sensation similar to actual reality by using a haptic device.

(R4 -3)

Fiber optic sensor non-linearity is mentioned but not quantified. Readers may question why closed-loop force control was abandoned.

(A4 -3)

We thank the reviewer for raising this important point regarding the sensor characteristics and control strategy.

We would like to clarify that the fiber optic sensor used in this study (FS-V31M) is commercially designed for binary presence detection (threshold-based ON/OFF), rather than for continuous analog displacement measurement. Consequently, the sensor's analog output exhibits not only significant non-linearity (due to the inverse square law and surface reflectance) but also substantial unit-to-unit variability.

Because the non-linear profile varies unpredictably across different sensor units and target surface conditions, presenting a single quantitative graph could be misleading as it does not represent a universal model of the system.

To clearly convey these points to the readers, we have included an additional explanation in the Materials and Methods section.

**Previous manuscript (6)**
Although alternatives like fiber optic sensors exist, we found their position data showed significant nonlinearity, rendering them inadequate for the precise position control required in a closed-loop system. Therefore, pressure can be estimated through a system model and used for control.
**Revised manuscript (6)**
Although alternatives like fiber optic sensors exist, the fiber optic sensor (FS-V31M) is primarily intended for binary presence detection rather than continuous measurement. Consequently, their analog output exhibits significant non-linearity and variability depending on the specific device unit and surface conditions. These characteristics make it difficult to establish a consistent linear model required for precise closed-loop feedback. Alternatively, pressure can be estimated through a system model and used for control.

(R4 -4)

4) fMRI figures are purely visual. No effect size maps or confidence indicators are shown.

(A4 -4)

We appreciate the reviewer's suggestion regarding the visualization of effect sizes. We would like to clarify that the fMRI figures presented in the manuscript are T-statistic maps, where the color intensity directly corresponds to the T-value, serving as a visual proxy for the signal strength relative to noise.

To provide precise quantitative evidence without overcrowding the visual results with additional maps, we have reported all key statistical indicators—including peak T-values (representing effect magnitude), cluster sizes, and p-values—in [Table 2] and [Table 3]. We believe that this combination of standard T-maps for spatial localization and detailed tables for quantitative assessment provides the most accurate and readable representation of the data for this study.

(R4 -5)

Minor grammatical and stylistic issues throughout (e.g., redundant phrases, awkward transitions). Not critical but noticeable.

(A4 -5)

Thank you for pointing it out. I've corrected a few grammatical errors and awkward connections, as shown below.

**Previous manuscript (7)**
1) (1) Developing a multi-finger haptic display that provides VR with immersive and fMRI-compatibility with fMRI.<br>2) The outcomes are showed in Table 3.<br>3) Given the constraints of MR environments, we chose pneumatic for our haptic device due to their high fMRI-compatibility.<br>4) We developed VR content to evaluate the reality and immersion with the proposed haptic display. Building upon this, we have developed an improved pneumatic cylinder for our multi-finger haptic display, focusing significantly on compactness and sealing to ensure fMRI-compatibility.
**Revised manuscript (7)**

1) (1) Developing a multi-finger haptic display that ensures immersive VR experiences and fMRI-compatibility.
2) The outcomes are shown in Table 3.
3) Given the constraints of MR environments, we chose pneumatic actuators for our haptic device due to their high fMRI-compatibility.
4) To realize this system, we first engineered an improved pneumatic cylinder for our multi-finger haptic display, focusing significantly on compactness and sealing to ensure fMRI-compatibility. Subsequently, we developed VR content to evaluate the reality and immersion provided by the proposed display.

(R4 -6)

Experiment 1: n = 13 Experiment 2: n = 7,These are very small samples, especially given whole-brain analyses. Low power inflates effect sizes and false discovery risk. Especially problematic since no correction survives. Re-frame conclusions accordingly. Alternatively, restrict interpretation to within-subject descriptive patterns.

(A4 -6)

Considering the small number of subjects, we focused our interpretation on the observed phenomena themselves, strictly reflecting the data. We have thus reframed the conclusions to clearly state the statistical limitations and avoid any exaggeration of the results.

**Previous manuscript (8)**
Our empirical investigation, involving 2 experiments, provided insightful evidence on the significant role of tactile feedback in enhancing immersion within VR environments. The study revealed that the incorporation of haptic feedback led to increased activation in brain regions associated with immersion, thereby underscoring the potential of our haptic display in enriching the VR experience.
**Revised manuscript (8)**
As a pilot investigation, our empirical study provided preliminary evidence regarding the potential influence of tactile feedback on immersion within VR environments. Although the sample size was limited, the observed patterns suggest that the incorporation of haptic feedback may correspond with modulated activation in brain regions associated with immersion. These results demonstrate the feasibility of using our haptic display to objectively assess the VR experience.

(R4 -7)

Trumpet fingering task uses visually cued button presses while breath input is fixed. This task resembles a button-press paradigm more than natural interaction. The added value of a multi-finger haptic device is not strongly demonstrated over simpler input devices. Need stronger justification for why this task requires multi-finger force feedback or acknowledge task limitations.

(A4 -7)

We acknowledge that the simplified trumpet task resembles a button-press paradigm in terms of motor output. However, the critical contribution of the multi-finger haptic display lies in its ability to provide realistic pneumatic resistance and stiffness comparable to a physical instrument, rather than the binary tactile click of a standard input device. Unlike simple springs or buttons, our device renders continuous force feedback (1N-10N), allowing the user to feel the compliance and mechanical impedance of a valve. This specific kinesthetic realism is what drives the 'sense of presence' and sensorimotor immersion we aimed to measure, which cannot be replicated by simpler binary switches.

Nevertheless, we agree with your assessment that the specific nature of the trumpet task, which typically requires a constant force threshold, does not fully exploit the versatile capabilities of our multi-finger device. Acknowledging this limitation, we have explicitly stated in the Limitation section that future research utilizing a broader range of dynamic tasks is necessary to demonstrate the device's advantages.

**Previous manuscript (9)**
**Revised manuscript (9)**
It should also be noted that the trumpet task resembles a button-press paradigm. However, our device distinguishes itself by providing realistic pneumatic resistance rather than the binary click of standard input devices. We recognize that this specific task did not fully exploit the device's variable force capabilities, necessitating future research with more dynamic tasks.
